# Relationships between GPS-derived outdoor activity space ambient heat exposure, mental health, and salivary cortisol in a longitudinal, repeated measures sample of adult Detroiters

**DOI:** 10.21203/rs.3.rs-9734906/v1

**Published:** 2026-06-05

**Authors:** Amber L. Pearson, Monika A. Tomaszewska, Catherine D. Brown, Rachel T. Buxton, Teresa H. Horton, Julie A. Perez, Karin A. Pfeiffer, Geoffrey M. Henebry

**Keywords:** urban heat, stress, biomarker, socio-economic status, green space

## Abstract

Exposure to heat in urban settings is a significant and growing public health concern. Yet, few studies have longitudinally measured everyday heat exposure using GPS-derived activity space data, paired with fine resolution satellite-derived heat measures, and no studies have measured effects on salivary cortisol in a community-dwelling sample. We address these gaps using data collected 2019–2023 across 11 neighborhoods in Detroit. Participants wore a GPS device and accelerometer over a one-week period. They completed a survey on demographics and mental health (anxiety and depression symptoms) and provided salivary cortisol samples (used to calculate diurnal slope) each year. We used a novel land surface temperature fusion product from ConstellR at daily cadence and 30-m spatial resolution to calculate heat exposure. We linked the seasonal time series of 2-band Enhanced Vegetation Index values calculated from the Harmonized Landsat-Sentinel-2 product with accumulated growing degree-days calculated from the ConstellR fusion product to model the land surface phenology at each pixel and used these modeled EVI2 values to estimate the exposure to greenness. We then tested whether higher heat exposure during routine daily outdoor activities was associated with poorer mental health or salivary cortisol data and whether these effects were attenuated by exposure to greenness. Among participants with ≥ 60 minutes of outdoor non-vehicle time, we observed a positive association between heat and anxiety (coef = 0.007, p-value = 0.062) and depression (coef = 0.007, p = 0.036) symptoms, after full confounder adjustment. Greenness was not independently, significantly associated with mental health measures and did not alter the effect size of heat exposure. We also observed a significant, positive association between heat exposure and cortisol slope (coef < 0.001, p-value = 0.029), indicative of higher stress. The consistency in our findings strengthens the evidence of the mental health impacts of everyday heat exposure, beyond extreme heat events. These findings have urban planning implications for increasing tree canopy, lowering impervious surfaces, providing heat shelters, and other urban cooling strategies. City-level or neighborhood-level heat action plans may help reduce the broader health impacts of rising temperatures in a warming climate.

## Introduction

Of myriad environmental threats facing urban populations, exposure to heat has been identified as a current and growing concern, especially during heat waves [1–4]. The urban heat island (UHI) effect is an intrinsic consequence of concentrations of impervious surfaces with enough mass to absorb sunlight that is later re-radiated in thermal infrared. The UHI has been recognized, characterized, modeled, and studied for decades [5–8], and the related satellite-derived metric, called the surface urban heat island effect, is now receiving considerable attention [9–12]. However, it is not impervious surfaces alone that affect heat experiences in cities. A landmark intercomparison study of 32 urban land surface models found inclusion of vegetation in the model captured latent and sensible heat fluxes across the year [13]. Thus, vegetation plays a central role in regulating the thermal environment of urban neighborhoods.

Most health studies focus on extreme heat events and mental health (e.g., [14]). Moreover, there is increasing evidence across countries that non-extreme ambient temperatures affect mental health outcomes (e.g., depression, anxiety, and psychological distress). For example, a study using UK Biobank data found harmful effects of UHI on mental health in middle-aged and older adults [15]. Another study linking ambient temperatures (not just extremes) to Emergency Department (ED) visits for mental illness, suicides, and self-reported days of poor mental health across the U.S., found that hot days increased all three admissions [16]. Notably, this study found no evidence of adaptation over time or moderation by air conditioning at home [16]. A recent longitudinal study from Thailand found that a 1°C increase in local temperature fluctuations was associated with higher psychological distress [17].

Both the heat exposures and their effects are disproportionately felt by older adults, low-income populations, and minoritized residents. The mechanisms appear to differ across these subpopulations, as some face greater exposure [18] (living in hotter neighborhoods with less greenspace) and others face greater sensitivity (pre-existing conditions, lower thermoregulation [19]). A recent systematic review concluded that the effects of extreme heat were consistently harmful for older adults’ mental well-being, including social isolation, cognitive dysfunction, affective disorders, and sleep disturbances [20]. Evidence from the Health and Retirement Study found that high exposure to extreme heat was associated with faster cognitive decline for non-Hispanic Black participants and residents of low-income neighborhoods, but not for White or Hispanic participants or those residing in higher income neighborhoods [21]. Finally, a UK survey of almost 1,500 participants reported high levels of negative wellbeing during a heat wave, with higher age and greater distance from greenspace predicting higher vulnerability. Still, a recent review concluded that more research is needed to contextualize the level to which greenspace may mitigate the adverse mental health effects of heat [22].

In theory, exposure to heat can affect mental health through intertwined physiological and psychological pathways. High temperatures place strain on the body’s thermoregulatory systems, activating the hypothalamic–pituitary–adrenal (HPA) axis, leading to the release of cortisol, a key stress hormone [23]. While short-term cortisol elevations can be adaptive, repeated or prolonged heat exposure may dysregulate this system, resulting in chronically elevated cortisol levels and flattened diurnal slopes [24, 25]. Sustained HPA-axis activation and elevated cortisol are associated with sleep-wake disruption and with anxiety/depressive symptoms [26, 27].

However, the mechanisms behind the heat-mental health relationship are not settled. A recent review found only limited evidence for HPA-axis activation, with increased psychological stress, reduced exercise, and sleep disruption as mediators [28]. Moreover, evidence of the heat-cortisol relationship is primarily derived from controlled lab studies rather than real-world conditions. In lab studies, even relatively small changes in ambient temperatures resulted in elevated cortisol [29] which were associated with higher perceived thermal discomfort [30].

Over the past 15 years, Detroit has been a changing urban landscape due, in part, to a policy of residential demolitions following massive population declines. The demolition policy was intended as a step toward urban redevelopment and neighborhood safety [31–33]. The demolitions have generated more than 25 square miles (65 km^2^) of vacant land within the City of Detroit. This high level of vacancy has resulted in some residents feeling isolated, fearful of crime, and stressed [34]. Recent funding, called the Detroit Neighborhood Beautification Program (NBP), has supported a community-led land use rehabilitation program designed to improve vacant lots. These projects involve garden creation, clean-up efforts (removal of litter and overgrown vegetation), and/or other improvements to urban spaces. More than 250 projects have been funded through 2025, with > 90 already completed.

Our study aimed to address gaps in knowledge related to the effects of ambient heat on mental health in a sample of predominantly low-income, minoritized residents. We leveraged device-derived measures of everyday mobility patterns collected annually over six years across 11 neighborhoods in Detroit as part of an NIH-funded project called the Study of Active Neighborhoods in Detroit (StAND). StAND monitored health and mobility of residents living near 11 city parks to detect changes in residents’ health over time [35]. In this study we aimed to bring cutting-edge remote sensing data and geospatial modeling to the GPS-derived outdoor activity space locations and mental health measurements collected for the StAND project. We quantified individual heat exposures during everyday activities (using GPS data) from Land Surface Temperature (LST) and to greenery from the 2-band Enhanced Vegetation Index (EVI2)We tested whether 1) higher exposure to urban heat during routine daily outdoor activities was associated with poorer mental health (i.e., anxiety and depression symptoms); 2) whether the association was also observed using a biomarker of stress - salivary cortisol and; 3) whether the effects of urban heat on mental health were attenuated by exposure to greenness.

## Methods

### Sample

The sample consists of adult residents (≥ 18 years old) living in neighborhoods surrounding selected parks in Detroit, Michigan. Recruitment occurred through mailings, community outreach (e.g., booths), and door-to-door contact [36]. Only one adult per household was enrolled. Participants were English-speaking and without mobility limitations. Enrollment occurred 2018–2022 (although there was no enrollment in 2020 due to COVID-19 research restrictions), sampled from 11 low-income Detroit neighborhoods.

#### Human ethics and consent to participate

All participants consented in writing, through approval by Michigan State University’s Institutional Review Board (#STUDY00002290) and in accordance with the Declaration of Helsinki.

#### Mental health measures

Mental health measures were captured each wave of data collection at the same time that the GPS and accelerometer devices were worn. Self-reported mental health measures included symptoms of anxiety and depression. Anxiety and depressive symptoms were measured via NIH’s Adult PROMIS-29 Profile v2.0 [37, 38]. T-scores for anxiety and depressive symptoms were generated by comparing values to the online tool reference population, the 2000 general US census population (mean = 50, standard deviation = 10), whereby lower t-scores indicate more favorable outcomes. The measures of anxiety and depressive symptoms have shown high internal consistency (Cronbach’s α > 0.88) [39]. Anxiety data were available for 1079 person-waves (n = 569 unique participants), and depression data were available for 1076 person-waves (n = 573 unique participants). In this analytical sample, consistency was high (depression Cronbach’s α = 0.923 and anxiety Cronbach’s α = 0.904).

In addition, we obtained salivary cortisol data as a biomarker of stress. Saliva samples were collected using the passive drool method [40] at waking and prior to going to sleep at night to permit calculation of time point concentrations and the slope of cortisol from waking to evening. Participants were asked to record the precise time when they awoke and when they took each sample. Samples were immediately frozen upon collection and kept frozen between − 72 and − 80C until analysis. Samples were then thawed, centrifuged and the supernatant aliquoted into replicate samples in smaller tubes and analyzed in duplicate. All samples were analyzed after data collection was complete to prevent issues related to inter-assay variability. Cortisol was measured using the Salimetrics ELISA kit as per manufacturer’s instructions [40]. Low and high concentration controls were prepared at analysis. Cortisol slope (i.e., diurnal decline) was then calculated as the difference between evening and waking cortisol concentration, divided by the number of hours between samples [41]. As a quality control step, we only utilized data that met the following criteria: the first or “waking” sample was taken within 10 minutes of when a person awoke and the final or “bedtime” sample was taken more than 12 hours but less than 16.5 hours after waking. Cortisol slope data were available for 217 person-waves (164 unique participants).

#### Remote sensing of land surface temperature – heat exposure measure

We used the ConstellR LSTfusion [42], proprietary land surface temperature (LST) fusion product that integrates observations from multiple satellite sensors across different spatial resolutions and overpass times into a spatially consistent dataset at 30-m resolution with daily measures expected at 1100 local solar time. The daily LST rasters from 2018 through 2023 were provided by ConstellR through special arrangement and they had already been subsetted to the greater Detroit area. All LST values were converted from Kelvin to degrees Celsius and reprojected to match Albers Equal-Area Conic projection and grid of the USGS National Land Cover Dataset [43]. To attenuate short-term noise while preserving temporal patterns, the LST time series were smoothed using a Savitzky–Golay filter [44] with a window size of 11 and a second-order polynomial. These filtered LST data provided the time series for estimating the daily heat exposures.

#### Remote sensing of the vegetated land surface – greenness exposure measure

NASA’s Harmonized Landsat–Sentinel-2 (HLS) is a unified Level-2 surface reflectance dataset that combines observations from the Operational Land Imager onboard NASA/USGS Landsat 8 and 9 satellites with the Multi-Spectral Instrument onboard ESA’s Copernicus Sentinel-2A, 2B, and 2C satellites. All observations are gridded to the common resolution of 30-m [45].

Using NASA’s AppEEARS (Application for Extracting and Exploring Analysis Ready Samples) online tool (https://appeears.earthdatacloud.nasa.gov/), we requested six bands from HLS product. Specifically, we selected the red, NIR, and quality bands for Landsat and Sentinel-2 covering the greater Detroit area from 2018 through the end of 2023. We then calculated the two-bands Enhanced Vegetation Index (EVI2) [46] using [Equation 1]:

[1] $$\text{EVI}2=2.5\times \frac{\left(NIR-RED\right)}{\left(NIR+2.4*RED+1\right)}$$

We applied the quality bands to mask out pixels flagged “Cloud” (bit 1) and “Cloud shadow” (bit 3). Additionally, we filtered any EVI2 values below 0 and above 1.2. All images were then reprojected to match the NLCD’s AEA projection and grid.

EVI2 observations from HLS were not available for every observation day during the study period. Thus, we estimated the daily greenness dynamics by growing the EVI2 as a function of growing degree-days (GDD), which can be interpreted as a proxy for insolation and thermal conditions relevant to the growth and development of herbaceous vegetation in the temperate mid-latitudes [47, 48]. We calculated GDD from the processed ConstellR LSTfusion image series using two different base temperatures (T_base_) of °C and 4°C as follows [Equation 2]:

[2] $$GDD_{t}=\text{max}\left(LST_{t}-T_{\text{base}},0\right)$$

Next, we accumulated growing degree-days (AGDD) to characterize the progression of thermal time over the course of the year as follows [Equation 3]:

[3] $$\text{AGD}D_{t}=\text{AGD}D_{t-1}+GDD_{t}$$

Next, we combined ConstellR-derived AGDD with HLS EVI2 and applied a downward-arching convex quadratic (CxQ) function to model the land surface phenology (LSP). We used a modified LSP [49] algorithm developed by [50], and subsequently revised [51] as follows [Equation 4]:

[4] $$\text{EVI}2=\alpha +\beta \times \text{AGDD}+\gamma \times AGDD^{2}$$

To reduce noise, snow impact, and spurious observations prior to model fitting, EVI2 values below 0.18 and AGDD values below 300 were excluded. In total, six model runs were performed per pixel–year combination, corresponding to two ConstellR AGDD datasets with different base temperatures and three Savitzky–Golay (SG) smoothing configurations applied to EVI2 (window sizes of 11, 13, and 15 with a second-order polynomial). For each LSP run, the parameter coefficients fitted for the intercept (α), linear term (β), and quadratic term (γ) were retrieved and used to model a seasonal progression of daily EVI2 values.

Finally, all modeled daily EVI2 outputs were combined into a single final layer, using the AGDD dataset with a base temperature of 4°C and smoothed EVI2 with SG window size of 11 as the reference layer. Missing observations from failed fits were subsequently filled in a hierarchical manner, first using results derived from AGDD with T_base_ = 4°C and SG window sizes of 13 and 15, followed by AGDD with T_base_ = 0°C and SG window sizes of 11, 13, and 15. This procedure ensured that the final EVI2 time-series represented the most complete and smoothed phenological trajectory for each pixel and year from which to estimate individual exposures to greenness.

### GPS-derived outdoor activity space data

To assign heat and greenness exposures to individuals, we linked environmental data with GPS mobility data based on space and time. Participants wore GPS devices (Canmore GT-730FL-S) and accelerometers (ActiGraph GT3x-BT, ActiGraph LLC, Pensacola, FL) on elastic belts with the devices placed at the right hip, worn over a one-week period for each year of data collection. Both GPS and accelerometer devices were initialized to record data at 30Hz. Data were aggregated and exported to 5-second epochs (for the use of lux data only). These linked GPS-accelerometer data were classed as indoor or outdoor using a previously validated rule-based point cluster-based method [52]. Point clusters were identified using a space-time clustering software (source codes are available on https://github.com/zackrzot/GPSAS_Destinations). Point clusters (within a 30-m radius) for a long enough duration (> 30 minutes), and/or low average speed (0.9m/s), or with over half of the cluster overlapping with a building and not in a park (as this would be an outdoor destination detected in our cluster software) were classified as indoor points. We defined outdoor points as those not within buildings and not within space-time clusters (deemed destinations) unless the destination was a park. Building footprint data were from the Southeast Michigan Council of Governments Open Data (Building Footprints, 2020 dataset), with subsequent annual validation by in-field assessment of all structures within the 11 study neighborhoods by our study team. Park location data were compiled from the DPRD. As a final step, accelerometer lux values (> 240) were also used to further distinguish outdoor from indoor points [53]. To exclude outdoor points which were spent in a vehicle, a series of rules were applied. First, we calculated the average speed of the linked GPS-accelerometer data at 1-minute aggregates. If the average speed was over 6.944 m/s (25 km/h) for a given minute, all GPS points within that minute were classified as in a vehicle. Next, to account for special cases (e.g., riding a bus that starts and stops frequently) we filtered points to verify that all points were properly classified using locations of bus routes, major roads, and parking lots. We also considered temporary vehicle slow-downs or stops by checking the classification and speed of surrounding minutes within the data. Ultimately, only linked GPS-accelerometer points that were classified as outdoor and non-vehicle were linked to environmental exposure data.

In total, we obtained 46,528,314 outdoor linked GPS-accelerometer coordinates/points collected from 840 person-waves (n = 442 unique participants). For every outdoor, non-vehicle GPS location, we generated heat exposure and greenness exposure. For anxiety and depression symptoms analyses, we utilized GPS data over the seven days prior to measurement of mental health and for salivary cortisol analyses, we used data from the 24h preceding salivary cortisol sampling. These values were then aggregated to generate values per hour of exposure.

To link these data, GPS points were reprojected to match the reference NLCD grid and spatially joined to grid cells. Within each grid cell, consecutive points for each participant, track, and date were grouped into subtracks by identifying gaps longer than 5 seconds and assigning continuous sequences a unique subtrack ID. For each subtrack, we calculated the number of points, start and end times, and total duration, which represents the time (in hour units) a participant spent in that grid cell during the continuous sequence of points. These summaries were then aggregated per participant, grid cell, and date. For each date or hour, corresponding environmental variables were extracted and were weighted by the adjusted total duration for each GPS track to quantify daily exposures to urban heat and greenness for each participant. Finally, we summed the exposures over the previous seven days and the previous 24 hours (available for n = 469 person-waves, n = 313 unique participants).

#### Confounder data

We included the following potential confounders: perceived safety walking in neighborhood during day, sex, race (Black or non-Black), employment (employed or not), and marital status (married/partnered or not). Feelings of safety in this study were measured using a single self-reported survey item assessing perceived neighborhood safety during daytime walking. Participants were asked to rate their agreement with the statement: “The crime rate in my neighborhood makes it unsafe to go on walks during the day,” using a 5-point Likert-type scale, where higher values indicate feeling unsafe. This measure is drawn from prior neighborhood environment surveys and similar items assessing perceived safety in past research [34, 54]. Finally, we also included the total minutes each participant spent outdoors, derived from the linked GPS-accelerometer wear time.

#### Primary statistical analyses

We conducted a longitudinal repeated-measures analysis of the 469 person-waves for which we had heat and greenness exposures. To understand the relationships between exposures to these environments and participants’ mental health, we fitted multilevel regression models to test for associations between heat exposure during routine daily outdoor activities with poorer mental health outcomes (i.e., perceived stress and anxiety and depression symptoms) separately. We adjusted for demographic confounders and perceived neighborhood safety while walking as potential confounders. We included a random effect for both wave and participant, to account for within-wave and within-person correlations.

### Sensitivity analyses

In planned sensitivity analyses we further verified 1) whether the effects of urban heat on mental health were attenuated by exposure to green space and; 2) if this association could be observed using salivary cortisol data (only available for a subset of the sample). Because having depression symptoms may lower your time spent outdoors and thus lower your heat exposure, we also tested our primary analyses on a subset of person-waves with at least 60 minutes spent outdoors over a one-week period. This threshold was selected to align with the 20-5-3 rule which states: people should aim to spend 20 minutes outdoors three times a week; spend five hours a month in semiwild outdoor locations; and spend three days a year in nature [55]. In the first sensitivity analysis, we fitted the fully adjusted primary regression models but also included the measure of greenspace exposure as an independent variable. We also fitted a regression model with the cortisol slope as the dependent variable and included all the same potential confounders that were used in the primary analyses.

## Results

In this sample of 469 person-waves for anxiety and depression analyses, we observed that those exposed to high levels of heat were significantly older, male, and unemployed (Table 1). This group spent significantly more time outdoors and were exposed to significantly more greenness. We did not observe significant differences across strata for anxiety or depression symptoms.

In regression modeling of our primary outcomes, we did not observe a significant, positive association between heat exposure and anxiety on the full sample (Table 2, Model 1). But, when we restricted to a sub-sample of participants with at least 60 minutes of outdoor, non-vehicle time, we observed a positive association which was marginally significant (Table S1, Model 1, coef = 0.007, p-value = 0.062), after full confounder adjustment. In depression models at the p < 0.10 level, both the full sample (Table 2, Model 3, coef = 0.007, p = 0.056) and the restricted sample (Table S1, Model 2, coef = 0.007, p = 0.036) yielded significant, positive associations between heat exposure and depression symptoms. In other words, for every one unit higher exposure to daily heat across a week, we would expect a 0.007 increase in anxiety and depression symptoms. Other factors were also significantly associated with these mental health outcomes, including feelings of being unsafe walking in the neighborhood, age, and female sex. When we included greenness exposures in the model, all significance levels for associations between heat and mental health became non-significant but the effect sizes remained similar (Table 2, Models 2 and 4). Greenness was not independently, significantly associated with either mental health measure. Exposure to greenness and exposure to heat were strongly correlated (r = 0.830, p < 0.001).

In our regression modeling of salivary cortisol slope in a subset of the sample for which biomarker data were available, we observed a significant, positive association between heat exposure and cortisol slope (coef = 0.0004, p-value = 0.029, Table 3). Negative slopes indicate a steep decline in salivary cortisol from waking to bedtime, which is a healthy response. Therefore, a positive association is indicative of higher stress.

## Discussion

In our longitudinal evaluation of heat exposure on mental health, we found that heat was significantly associated with poorer mental health and higher stress using biomarker data, after adjustment for a suite of confounders. Our findings were consistent across all three outcome measures, although with varying levels of statistical significance. Further, we did not find evidence that greenness exposure lowered the effects of heat on mental health. For both heat and greenness exposure, we used satellite-derived measures at fine spatial and temporal scales. We relied on outdoor non-vehicle linked GPS-accelerometer data to assign exposures, allowing us to more precisely evaluate participants’ real-time exposures. This approach allowed us to overcome two previous limitations noted in the heat-health literature. First, some prior studies have been limited by reliance on monitoring stations which yield coarse heat exposure estimates [56]. Second, extant research has also assigned heat exposure by date and residential location, not accounting for actual time spent outdoors [57].

The extant longitudinal evidence generally supports our findings that heat exposure can threaten mental health. A previous study from the China Health and Retirement Longitudinal Study reported that heat exposure days over one year significantly increased depression among adults (aged 45y+) [58]. Likewise, an ecological, U.S. state-level analysis found that higher extreme heat exposure was significantly, positively associated with the prevalence of mental health issues, regardless of state demographic factors [59]. While there are several studies of the mental health effects of heat, no studies to our knowledge specifically measured salivary cortisol in real-world conditions and tested for relationships with exposure to heat. Our study, thus, makes an important contribution to knowledge in this area for two reasons. First, biomarkers, such as salivary cortisol, may provide insights into how environmental exposures affect underlying biological mechanisms of stress. Second, measuring salivary cortisol in a non-controlled, real-world setting is critical to improve our understanding of how everyday heat exposures affect physiological stress during routine activities. Prior to our study, investigations of the effects of heat on cortisol were limited to laboratory and small experimental studies [29, 30, 60]. Our results echo these studies, but the real-world conditions of our study enhance the practical implications.

We did not detect the attenuation of the effect of heat on mental health when considering greenness. Greenness has frequently been proposed as a solution to reduce urban heat, as greenery has been shown to reduce surface temperature from 2–17°C depending on its location (i.e., surface greenery vs vertical greenery on buildings) [61]. Our findings may be related to the context of our study site. In Detroit, there are high levels of vacancy and thus greenness, which may not yield mental health benefits [62, 63] or may not yield benefits large enough to overcome those related to heat exposure. It is important to note that, in our study, there are very high levels of greenness in general and those exposed to high levels of heat were also exposed to high levels of greenness, making it difficult to untangle these relationships. This is likely due to the ubiquitous nature of vacant lots. It is unclear from our analysis whether heat and greenness exposure actually occurred in the same location and time, but rather were both high or low when aggregated across observation days for participants. Further, summer high temperatures in Detroit during the observation period ranged from 90F to 96F, with an average high below 85F. Future research in other settings is warranted, including cities with a wide variety of levels of greenness and summer temperatures and those that include both high quality (e.g., maintained or biodiverse [64]) greenspaces and lower quality greenspaces [65] to untangle the buffering effects on stress and mental health.

### Strengths and limitations

This study has several strengths, including the use of GPS-derived measures of outdoor activity spaces, which provide a more precise and individualized assessment of environmental exposures compared to static residential measures or self-reported time spent outdoors. The use of linked GPS-accelerometer data also permitted the spatial and temporal linkage to heat and greenness exposures. The longitudinal design strengthens causal inference by capturing changes over time, and the inclusion of a biomarker of stress offers an objective complement to self-reported mental health outcomes. Findings were consistent across multiple outcome measures, enhancing confidence in the robustness of the results, and were supported by high-quality satellite data that improved exposure assessment. However, some limitations should be considered. The study did not account for conditions within the home environment, such as air conditioning access or energy costs, which may modify heat exposure and related stress. Additionally, the sample consisted of older adults, which may limit generalizability to younger populations; however, this focus is also a strength, as older individuals are more vulnerable to the adverse health effects of heat exposure.

### Future research and policy implications

Building on these findings, several future research directions emerge. Future studies could usefully examine heterogeneity in effects across sub-populations, including younger individuals and socioeconomically vulnerable groups to better understand differential susceptibility to everyday heat exposure. Incorporating indoor environmental conditions (e.g., access to air conditioning, housing quality, and energy affordability) would provide a more complete picture of total heat exposure or mitigation. Future work could also explore potential mediating pathways, including sleep disruption, social behavior changes, and cumulative physiological stress indicators (e.g., allostatic load). Studies over a longer observation period (more than seven days) may also provide insights in the longer term mental health trajectories and heat exposure. Expanding to diverse geographic and climatic contexts would help confirm or disconfirm our findings and strengthen generalizability.

These findings also have urban planning and health policy implications. Public health strategies should extend beyond a focus on extreme heat events to address the cumulative mental health burden of everyday heat exposure. Urban planning and climate adaptation policies could prioritize heat mitigation strategies such as increasing tree canopy, lowering impervious surfaces, providing heat shelters in areas with high pedestrian traffic, and implementing cool roofs and pavements. Housing policies that improve thermal comfort, particularly for vulnerable populations, through energy assistance programs and minimum housing standards may also be important but these factors are beyond scope for the current study. Finally, incorporating mental health considerations into heat action plans and early warning systems may help reduce the mental health burden of everyday exposures to heat.

## Conclusion

In our longitudinal sample relying on device-derived time spent outdoors data on mental health, salivary cortisol, and heat exposure, we found that heat was significantly associated with poorer mental health and higher stress using biomarker data, after adjustment for a suite of confounders. The consistency in our findings strengthens the evidence of the mental health impacts of everyday heat exposure, beyond extreme heat events. These findings have urban planning implications for increasing tree canopy, lowering impervious surfaces, providing heat shelters, and other urban cooling strategies. City-level or neighborhood-level heat action plans may help reduce the broader health impacts of rising temperatures in a warming climate.

## Supplementary Material

Supplementary Files

This is a list of supplementary files associated with this preprint. Click to download.

- PearsonHeatStressSM14May2026.docx

## Figures and Tables

**Table 1:** Sample descriptive statistics stratified by heat exposure

Characteristic	High heat	Low heat	Total	p-value
	*n = 235 person-waves*	*n = 234 person-waves*	*n = 469 person-waves*	
Age, mean (sd)	58.1 (13.2)	55.6 (15.2)	56.8 (14.3)	0.027
Female sex, %	50.2	63.7	56.9	0.003
Black, %	89.4	87.6	88.5	0.552
Employed, %	21.6	31.4	26.5	0.016
Depression symptoms, mean (sd)	53.3 (9.3)	52.0 (10.0)	52.7 (9.6)	0.163
Anxiety symptoms, mean (sd)	54.9 (10.0)	55.4 (10.0)	55.1 (10.0)	0.668
Minutes spent outdoors, mean (sd)	139.2 (75.3)	69.3 (43.8)	105.8 (71.3)	< 0.001
Greenness exposure, mean (sd)	2.8 (1.7)	0.7 (0.6)	1.8 (1.7)	< 0.001

**Table 2:** Regression modeling results for anxiety and depression symptoms

independent variables	Anxiety - Model 1			Anxiety - Model 2			Depression - Model 3			Depression - Model 4		
	coef	p-value	95%ci	coef	p-value	95%ci	coef	p-value	95%ci	coef	p-value	95%ci
heat exposure	0.004	0.228	−0.003–0.011	0.006	0.267	−0.006–0.015	0.007	0.056	−0.000–0.013	0.005	0.370	−0.006–0.015
greenness exposure				−0.234	0.661	−1.283–0.814				0.223	0.659	−0.765–1.210
female	2.284	0.023	0.317–4.252	2.284	0.023	0.317–4.252	2.247	0.017	0.396–4.098	2.254	0.017	0.403–4.104
age	−0.103	0.008	−0.179 - −0.027	−0.102	0.008	−0.178 - −0.027	−0.114	0.002	−0.185 - −0.04	−0.114	0.002	−0.185 - −0.042
employed	−0.329	0.782	−2.656–1.998	−0.387	0.746	−2.731–1.956	−0.369	0.744	−2.582–1.843	−0.316	0.781	−2.543–1.911
married/partnered	0.073	0.954	−2.428–2.575	0.176	0.892	−2.369–2.722	−0.632	0.597	−2.980–1.715	−0.723	0.553	−3.111–1.665
feeling unsafe walking	1.579	< 0.001	0.835–2.323	1.571	< 0.001	0.827–2.316	1.760	< 0.001	1.061–2.460	1.767	< 0.001	1.066–2.467
May	*reference*			*reference*			*reference*			*reference*		
June	1.157	0.492	−2.143–4.457	1.112	0.510	−2.194–4.418	0.937	0.557	−2.193–4.067	0.943	0.555	−2.189–4.075
July	4.088	0.022	0.594–7.582	4.048	0.023	0.550–7.546	3.149	0.065	−0.198–6.496	3.132	0.067	−0.213–6.487
August	−0.436	0.814	−4.077–3.205	−0.577	0.760	−4.280–3.125	−0.522	0.777	−4.139–3.094	−0.496	0.790	−4.151–3.159
September	2.635	0.168	−1.108–6.377	2.527	0.190	−1.249–6.303	2.925	0.134	−0.899–6.748	2.097	0.137	−0.924–6.739
October	4.424	0.039	0.217–8.631	4.359	0.043	0.142–8.575	3.353	0.132	−1.004–7.710	3.289	0.137	−1.051–7.630
November	1.890	0.649	−6.254–10.034	1.886	0.650	−6.256–10.028	4.405	0.272	−3.448–12.26	4.268	0.286	−4.574–12.110
time spent outdoors	0.002	0.811	−0.015–0.019	0.002	0.824	−0.015–0.019	−0.003	0.755	−0.018–0.013	−0.002	0.771	−0.018–0.013

**Table 3:** Regression modeling results for salivary cortisol slope

independent variables	coef	p-value	95%ci
heat exposure	< 0.001	0.029	0.000–0.001
female	−0.005	0.627	−0.027–0.017
age	< 0.001	0.514	−0.001–0.001
employed	−0.002	0.877	−0.028–0.024
married/partnered	−0.021	0.155	−0.051–0.008
feeling unsafe walking	0.002	0.728	−0.007–0.011
May	*reference*		
June	0.012	0.504	−0.024–0.049
July	0.009	0.610	−0.027–0.046
August	−0.020	0.271	−0.057–0.016
September	0.010	0.643	−0.031–0.050
October	−0.013	0.578	−0.06–0.033
time spent outdoors	<−0.001	0.093	<−0.001 – <0.001

## Data Availability

The StAND data that support the findings of this study are available from the lead author, but restrictions apply to the availability of these data, which were collected following guidance and approval from the Michigan State University IRB (#STUDY00002290) and so are not publicly available. The data are, however, available upon request and IRB approval.
